# The association between interhospital transfer and sepsis guideline adherence among rural emergency department patients

**DOI:** 10.1371/journal.pone.0351133

**Published:** 2026-07-10

**Authors:** Cole Wymore, Eliezer Santos León, Priyanka Vakkalanka, Uche Okoro, Karisa K. Harland, Brian M. Fuller, Kalyn Campbell, Morgan B. Swanson, Brett Faine, Anne Zepeski, Edith A. Parker, Luke J. Mack, Amanda Bell, Katie DeJong, Keith Mueller, Elizabeth Chrischilles, Christopher R. Carpenter, Kelli Wallace, Michael P. Jones, Marcia M. Ward, Nicholas M. Mohr

**Affiliations:** 1 Department of Emergency Medicine, University of Iowa Carver College of Medicine, Iowa, Iowa, United States of America; 2 Merck Sharp and Dohme, LLC, Rahway, New Jersey, United States of America; 3 Department of Anesthesiology, Division of Critical Care, Washington University School of Medicine, St. Louis, Missouri, United States of America; 4 Department of Emergency Medicine, Washington University School of Medicine, St. Louis, Missouri, United States of America; 5 Department of Surgery, Hennepin County Medical Center, Minneapolis, Minnesota, United States of America; 6 Department of Pharmaceutical Practice, College of Pharmacy, University of Iowa, Iowa, Iowa, United States of America; 7 Department of Community and Behavioral Health, University of Iowa College of Public Health, Iowa, Iowa, United States of America; 8 Avera eCARE, Sioux Falls, South Dakota, United States of America; 9 Department of Family Medicine, Sanford Health, Sioux Falls, South Dakota, United States of America; 10 Department of Health Management and Policy, University of Iowa Carver College of Medicine, Iowa, Iowa, United States of America; 11 Department of Epidemiology, University of Iowa College of Public Health, Iowa, Iowa, United States of America; 12 Department of Emergency Medicine, Mayo Clinic, Rochester, Minnesota, United States of America; 13 Department of Biostatistics, University of Iowa College of Public Health, Iowa, Iowa, United States of America; 14 Department of Anesthesia, Division of Critical Care, University of Iowa Carver College of Medicine, Iowa, Iowa, United States of America; Christian Medical College Vellore, INDIA

## Abstract

**Introduction:**

The objective of this study was to assess whether interhospital transfer delays completion of the Surviving Sepsis Campaign (SSC) 3-hour bundle among rural sepsis patients.

**Methods:**

We conducted a multicenter (n = 23) cohort study among adult rural sepsis patients in an ED-based telemedicine network between August 2016 and June 2019. The primary exposure was interhospital transfer and the primary outcome was completion of the SSC 3-hour bundle. Secondary outcomes included SSC component adherence, ED length-of-stay, and in-hospital mortality. We used a Cox proportional hazards model to evaluate time to bundle completion by transfer status, generalized linear models for dichotomous outcomes, and linear and quantile regression for ED length-of-stay.

**Results:**

A total of 1,191 patients were treated in participating hospitals, with 359 (30%) undergoing interhospital transfer. SSC bundle adherence was similar in both groups (difference 2.2%; 95% confidence interval [CI]: – 2.2, 6.5%). Among eligible patients, transfer was associated with a longer ED length-of-stay (3.1 hours vs 2.6 hours; difference 0.6 hours; 95%CI: 0.4, 0.7).

## Introduction

Sepsis is a life-threatening condition that has doubled in incidence over the past decade and has become a leading cause of United States in-hospital death [1]. Sepsis patients are commonly admitted from the emergency department (ED), and early aggressive ED care has been shown to improve survival [2,3]. The cornerstone of early sepsis care centers around the Surviving Sepsis Campaign (SSC) guidelines, which outline strategies for early recognition, appropriate antibiotic administration, and hemodynamic resuscitation [2,4–8]. This standardization in care has contributed to the 25% reduction in sepsis-related mortality over the past two decades, [3,9] and guideline adherence has been shown to be associated with improved survival [10]. Low-volume EDs, however, have 38% higher sepsis mortality than high-volume EDs, [11] and interhospital transfer does not completely ameliorate that risk [12]. Thus, rural sepsis patients represent a population for which additional attention and effort is needed in order to reduce disparities.

Rural EDs face several challenges in providing care to critically ill patients, [13–15] including staffing and familiarity with low-frequency conditions [16]. Several strategies have been adopted to improve rural outcomes, including performance improvement programs and regionalization. Regionalization, or the practice of transferring patients to higher capability hospitals, is commonly used in rural sepsis care, and several studies have shown care in high-volume hospitals to be associated with improved sepsis-related mortality [11,14,17]. Sepsis patients who transferred from low-volume to high-volume centers are still estimated to have 9% higher mortality compared to non-transferred patients receiving care entirely in high-volume centers, possibly due to delays in guideline-adherent care [12,18]. Therefore, the optimal regionalization strategy to improve sepsis survival for patients presenting to low-volume EDs is uncertain.

The time-consuming procedures associated with interhospital transfer may interfere with time-sensitive care and could harm patients. In a study analyzing EDs participating in the national Emergency Quality Network (E-QUAL), compliance with the sepsis quality measures of intravenous fluid resuscitation and antibiotic administration were lower in rural compared to urban EDs [19]. Since the quality measures studied were limited to ED care, this finding is likely a reflection of delays in care occurring in the ED (i.e., reconstituting antibiotics and administering fluids). Furthermore, there may not be enough time to address delays in patients undergoing interhospital transfer. The primary objective of this study was to test the hypothesis that interhospital transfer in rural ED sepsis patients reduces completion of the SSC 3-hour bundle, as a possible explanation for why interhospital transfer is not more effective at reducing mortality. Secondary objectives were to assess whether interhospital transfer is associated with individual elements of bundle adherence, ED length of stay (LOS), and in-hospital mortality.

## Methods

### Study design, setting, sources, and participants

This was a secondary analysis of a larger multicenter (n = 23) cohort study, the TELEmedicine as a Virtual Intervention for Sepsis care in Emergency Departments (TELEVISED) study—a study of patients with sepsis presenting to Midwestern rural EDs that participated in an ED-based telemedicine (tele-ED) network between August 2016 and June 2019. We have reported on the methods and results of the parent study previously [20,21].

Data acquisition for this study began on April 13, 2022. Data collection was performed using manual chart abstraction of medical records by three research assistants using the methods described in Kaji, et al. [22]. Identifiable individual data was available to abstractors but no identifiable data was abstracted for this study. Data abstractors received formal training on the data collection tools, and duplicate data collection was performed on 10% of randomly selected charts for quality control. Participants included all adult (age ≥ 18 years) sepsis patients seen in a participating rural ED during the study period. Rural EDs were defined by being in a county designated as rural by the Health Resources and Services Administration [23]. Sepsis was defined as requiring all of the following criteria: 1) inpatient discharge diagnosis of sepsis based on *International Classification of Diseases, 10th edition, Clinical Modification* (ICD-10-CM) codes, 2) infection diagnosed in the ED (based on clinical documentation), 3) organ failure in the ED (defined as a Sequential Organ Failure Assessment Score [SOFA] of 2 or greater or a change of at least 2 in those with chronic disease), and 4) at least 2 systemic inflammatory response syndrome (SIRS) criteria in the ED. Patients who were transferred out of the participating hospital network were excluded for missing data. Because this was a secondary analysis of the parent TELEVISED study, we included cases that were seen either with remote physician telemedicine consultation (in addition to local ED staff) or without physician telemedicine consultation, but we excluded cases that were treated with remote nurse consultation.

This manuscript is reported consistent with the Template for Intervention Description and Replication checklist for Population Health and Policy (TIDieR-PHP) and the study was designed in accordance with the Strengthening the Reporting of Observational Studies in the Epidemiology (STROBE) statement [24,25]. This project was approved under waiver of informed consent by the University of Iowa Institutional Review Board prior to study commencement.

### Exposures and outcomes

Our primary exposure was interhospital transfer (vs. local admission), and our primary outcome of interest was 3-hour SSC bundle completion. Patients were divided into two cohorts: 1) those who were transferred from the index hospital ED to another in-network hospital and 2) those admitted directly to an inpatient unit at the same hospital as the index ED. The primary outcome was complete adherence to all components of the SSC 3-hour bundle. Secondary outcomes included completion of individual components of the SSC 3-hour bundle, ED length of stay, and mortality.

### Definitions

Three-hour bundle completion was defined as cases that received all qualifying components of the SSC 3-hour bundle (measure lactate, draw blood cultures before antibiotics, administer appropriate broad-spectrum antibiotics, and administer 30 mL/kg crystalloid fluid bolus if lactate > 4 mmol/L or systolic blood pressure < 90 mmHg) within the first 3 hours of meeting all diagnostic criteria in the ED and prior to hospital admission or interhospital transfer [4]. Antibiotics were defined as broad-spectrum if they included coverage for methicillin-resistant *Staphlococcus aureus* (MRSA) and *Pseudomonas aeruginosa*. For time-to-completion analyses, time zero was defined as the initial set of vitals obtained in the ED that met systemic inflammatory response syndrome (SIRS) criteria [26].

### Analysis

Descriptive statistics were used to compare baseline characteristics. We expected that patients with higher illness severity would be more likely to be transferred, but they also may be more likely to have bundle adherence, so we used multivariable regression techniques to account for confounding by severity of illness. We included in our dataset demographic variables (e.g., age, sex), comorbidities, care-associated variables (e.g., level of ED provider training, mode of arrival), and severity of illness (e.g., SOFA score, infection source, surgical intervention, triage vital signs, ED vasopressor use, ED mechanical ventilation use). These variables were selected *a priori* for inclusion in the regression models. Telemedicine use was further included to account for confounding effect on patient’s treatment management. We screened for collinearity using the variance inflation factor.

We conducted our study using complete case analysis, and any cases with missing exposures or outcomes were excluded. For our primary outcome, completion of 3-hour SSC bundle, we used the Brown-Mood median test for differences between transferred and locally admitted groups [27]. Our primary model had an outcome of 3-hour SSC bundle adherence, then we developed similar models using the same covariates with the outcome of each of the elements of the 3-hour bundle to better understand the specific manner in which interhospital transfer was associated with each component of bundle adherence. For the time-to-adherence of the SSC 3-hour bundle, we used a Cox proportional hazards model, with right-censoring occurring at 180 minutes [28,29]. For dichotomous outcomes, we used generalized linear models with a binomial distribution and a logit link [30,31]. We used Firth logistic regression as a penalized approach for two dichotomous outcomes due to data sparsity issues [32–34]. For the continuous outcome of ED LOS, initial Q-Q plots indicated a violation of normality assumption for residuals. We therefore used both linear regression and quantile regression to the median for the continuous response variable [35]. We used a stratified analysis to conduct adherence measurement separately in those transferred above and below the median amount of time during interhospital transfer. All effects were reported as adjusted hazard ratios (aHR), odds ratios (aOR), or beta coefficients in linear and quantile regressions with 95% confidence intervals (CIs), and a two-tailed p-value of less than 0.05 was considered significant. All analyses were conducted using R, version 4.5.1 (R Foundation, Vienna, Austria).

## Results

**Fig 1:**
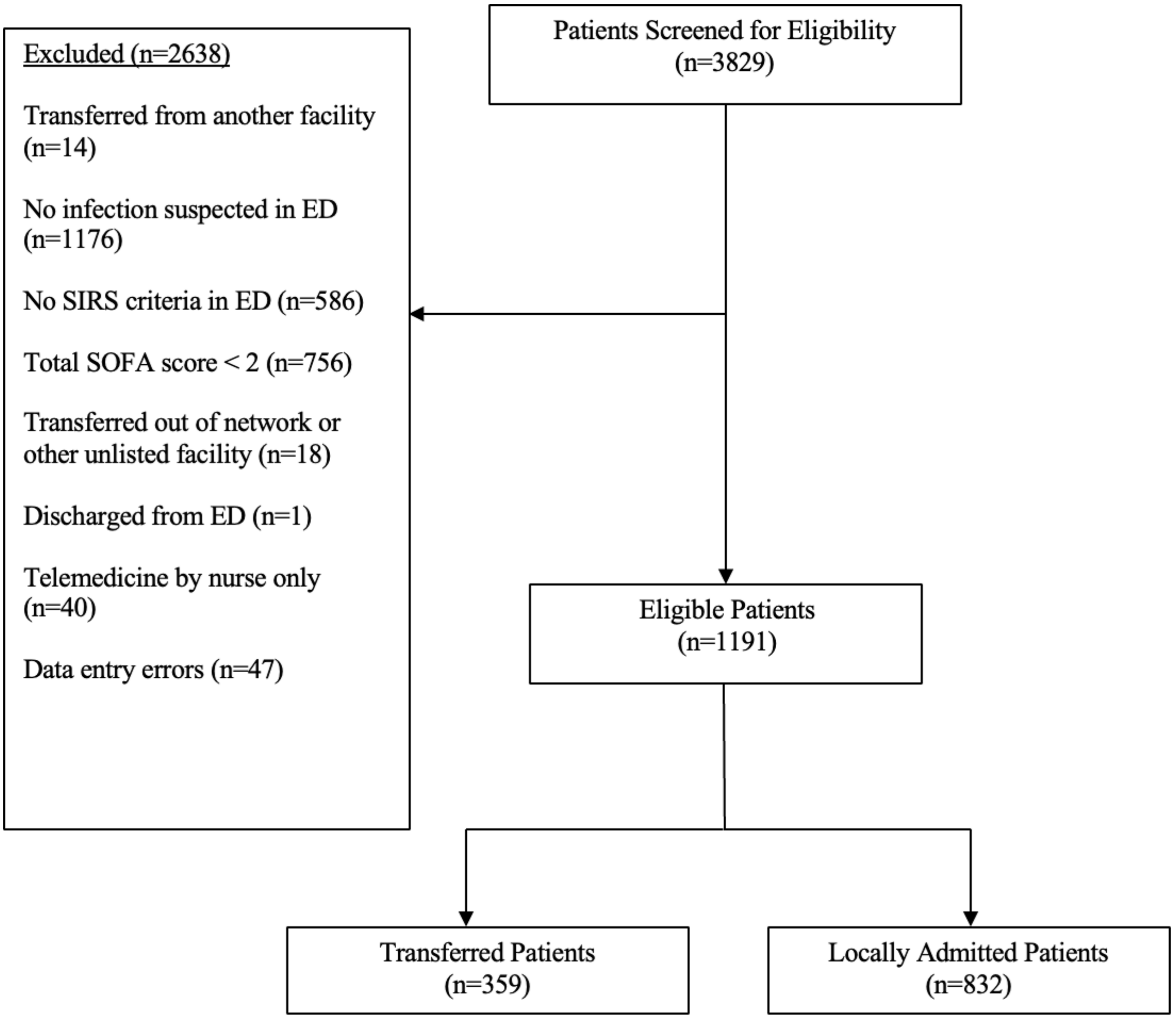
Flow chart of participants.

A total of 1,191 patients were included in the analysis, of which 359 (30%) were transferred from the rural ED to another hospital. The most common reasons for exclusion were not meeting SIRS criteria in the ED and no ED provider documentation of suspected infection (Fig 1). Patients were predominately male (55%) and between the ages of 60–89 years (73%). The most common source of infection was respiratory, which was identified in 635 (53%) patients. Of the included patients, 586 (49%) had a SOFA score of 2–3 and 537 (45%) had a score of 4–7. (Table 1) shows additional characteristics of the study population.

**Table 1:** Characteristics of participants.

	All Cases (n = 1191) n (%)	Admitted to local Hospital (n = 832) n (%)	Transferred (n = 359) n (%)	Difference (95% CI)
**DEMOGRAPHICS**				
Age^a^				
<30 y	10 (0.8)	6 (0.7)	4 (1.1)	−0.4 (−1.6, 8.2)
≥30 y and <40 y	41 (3.4)	25 (3.0)	16 (4.5)	−1.5 (−3.9, 9.8)
≥40 y and <50 y	68 (5.7)	34 (4.1)	34 (9.5)	−5.4 (−8.7, −2.1)
≥50 y and <60 y	121 (10.2)	72 (8.7)	49 (13.7)	−5.0 (−9.0, −0.1)
≥60 y and <70 y	306 (25.7)	197 (23.7)	109 (30.4)	6.7 (1.1, 12.3)
≥70 y and <80 y	272 (22.8)	188 (22.6)	84 (23.4)	0.8 (−4.4, 6.0)
≥80 y and <90 y	286 (24.1)	233 (28.0)	53 (14.8)	−13.2 (−18.0, −8.5)
≥90 y	87 (7.3)	77 (9.3)	10 (2.8)	−6.5 (−9.1, −3.9)
Female	536 (45.0)	390 (46.9)	146 (40.7)	−5.3 (−10.5, 0)
Body mass index, kg/m^2^				
<18.5	42 (3.5)	32 (3.9)	10 (2.8)	−1.6 (−3.2, 1.1)
≥18.5 and < 25	294 (24.7)	216 (26.0)	78 (21.7)	4.2 (−0.01, 9.4)
≥25 and <30	300 (25.2)	210 (26.9)	90 (27.1)	0 (−5.2, 5.5)
≥30 and <35	204 (17.1)	140 (16.8)	64 (17.8)	1.0 (−3.7, 5.7)
≥35	274 (2.0)	184 (22.1)	90 (25.1)	3.0 (−2.3, 8.3)
Missing	77 (6.5)	50 (6.0)	27 (7.5)	−1.5 (−4.7, 1.7)
Latinx	14 (1.2)	9 (1.1)	5 (1.4)	0.3 (−1.1, 1.7)
Race				
White	1059 (88.9)	742 (89.2)	317 (88.3)	−0.9 (−4.8, 3.1)
African American		3 (0.4)	5 (1.4)	1.0 (−0.3, 2.3)
American Indian or Alaska Native	8 (0.7) 93 (7.9)	64 (7.7)	29 (8.1)	0.4 (−3.0, 3.7)
Other	3 (0.3)	1 (0.1)	2 (0.6)	0.4 (−0.4, 1.2)
Missing	28 (2.4)	22 (2.6)	6 (1.7)	−1.0 (−2.7, 0.7)
**PATIENT CHARACTERISTICS**				
Triage Temperature, C,				
<37	426 (35.8)	270 (32.5)	156 (43.5)	11.0 (5.0, 17.0)
≥37 and < 37.5	155 (13.0)	109 (13.1)	46 (12.8)	−0.3 (−4.4, 3.9)
≥37.5 and <38	144 (12.1)	116 (13.9)	28 (7.8)	−6.1 (−9.8, −2.5)
≥38	380 (31.9)	290 (34.9)	90 (25.1)	−9.8 (−15.3, −4.3)
Missing				
Triage Mean arterial blood pressure, mmHg,				
<80	333 (28.0)	224 (26.9)	109 (30.4)	3.4 (−2.2, 9.1)
≥80 and < 90	201 (16.9)	150 (18.0)	51 (14.2)	−3.8 (−8.3, 0)
≥90 and <100	221 (18.6)	166 (20.0)	55 (15.3)	−4.6 (−9.2, 0)
≥110	369 (30.0)	251 (30.2)	118 (32.9)	2.7 (−3.1, 8.5)
Triage systolic blood pressure, mmHg,				
<115	341 (28.6)	224 (26.9)	117 (32.6)	5.7 (0, 11.4)
≥115 and < 130	217 (18.2)	160 (19.2)	57 (15.9)	−3.4 (−8.0, 1.3)
≥130 and <150	291 (24.4)	206 (24.8)	85 (23.7)	−1.1 (−6.4, 4.2)
≥150	275 (23.1)	201 (24.2)	74 (20.6)	−3.6 (−8.6, 1.6)
Missing	67 (5.6)	41 (4.9)	26 (7.2)	2.3 (−0.7, 5.4)
SOFA Score				
2–3	586 (49.2)	466 (56.0)	120 (33.4)	−22.6 (−28.5, −16.7)
4–7	537 (45.1)	345 (41.5)	192 (53.5)	12.0 (5.9, 18.2)
≥8	68 (5.7)	21 (2.5)	47 (13.1)	10.6 (6.9, 14.2)
Source of Infection				
Respiratory	635 (53.3)	484 (58.2)	151 (42.1)	−16.1 (−22.2, −10.0)
Genitourinary	211 (17.7)	157 (18.9)	54 (15.0)	−3.8 (−8.4, 0.7)
Abdominal	77 (6.5)	43 (5.2)	34 (9.5)	4.3 (0.9, 7.7)
Skin/Soft Tissue	109 (9.2)	69 (8.3)	40 (11.1)	2.9 (−0.9, 6.6)
Meningitis	14 (1.2)	6 (0.7)	8 (2.2)	1.5 (−0.1, 3.1)
Unknown/Other	145 (12.0)	73 (8.9)	72 (20.1)	11.2 (6.7, 15.9)
Surgery during hospital stay	81 (6.8)	39 (4.7)	42 (11.7)	7.0 (3.4, 10.6)
Telemedicine Use	326 (27.4)	40 (4.8)	286 (79.7)	74.9 (70.5, 79.3)
Mechanical ventilation in ED	108 (9.1)	38 (4.6)	70 (19.5)	14.9 (10.6, 19.3)
Vasopressor support in ED	134 (11.3)	61 (7.3)	73 (20.3)	13.0 (8.5, 17.5)
Provider Type				
Physician	1012 (85.0)	762 (91.6)	250 (69.6)	−22.0 (−27.1, −16.8)
APP with physician	116 (9.7)	54 (6.5)	62 (17.3)	10.8 (6.5, 15.0)
APP alone	63 (5.3)	16 (1.9)	47 (13.1)	11.2 (7.6, 14.8)
**COMORBIDITIES**				
COPD	378 (31.7)	279 (33.5)	99 (27.6)	−6.0 (−11.6, 0)
Cirrhosis	31 (2.6)	17 (2.0)	14 (3.9)	1.9 (0, 4.1)
Solid organ transplant	12 (1.0)	8 (1.0)	4 (1.1)	0 (−1.1, 1.4)
Cancer	354 (29.7)	254 (30.5)	100 (27.9)	−2.7 (−8.3, 2.9)
Diabetes	440 (36.9)	310 (37.3)	130 (36.2)	−1.1 (−7.0, 4.9)
Chronic Dialysis	45 (3.8)	14 (1.7)	31 (8.6)	7.0 (3.9, 10.0)
Asthma	143 (12.0)	96 (11.5)	47 (13.1)	1.6 (−2.6, −5.7)
Hypertension	856 (71.9)	615 (73.9)	241 (67.1)	−6.8 (−12.5, −1.1)
Chronic heart failure	255 (21.4)	175 (21.0)	80 (22.3)	1.3 (−3.9, 6.4)
HIV	1 (0.1)	1 (0.1)	0 (0)	−0.1 (−0.4, 0.1)

^a^Abbreviations: y (years).

### Patient characteristics

Relative to transferred patients, locally admitted patients were more likely to be 80 years or older (37% vs 18%; difference 19.7%, 95%CI: 14.4, 25.0%), had physician provided care (92% vs 70%; difference 22%, 95%CI: [−27.1], [−16.8]), and had lower severity of illness (SOFA score between 2 and 3; 33% transferred vs. 56% locally admitted; difference 22.6%; 95%CI: 16.7, 28.5%). More locally admitted patients were identified as having respiratory source of infection (58% vs 42%; difference 16.1%; 95%CI 10.0, 22.2%), while more transferred patients had an unknown source of infection (20% vs 9%; difference 11.2%; 95%CI: 6.7, 15.9%). Transferred patients were more likely to require mechanical ventilation (20% transferred vs 5% non-transferred; difference 14.9%; 95%CI: 10.6, 19.3) and vasopressor therapy (20% vs 7%; difference 13.0%; 95%CI: 8.5, 17.5) in the ED.

### Bundle adherence

Transferred and locally admitted patients had similar median time to 3-hour bundle completion (55 vs 50 min; difference 4 min; 95%CI: −3, 14) (Table 2). Completion of the SSC 3-hour bundle was similar between transferred and locally admitted patients (12.3% vs. 14.4%; difference −2.2%; 95%CI: [−6.5], 2.2%). Adjusted for potential confounders, the event rates of SSC 3-hour bundle completion were not significantly different for these groups (aHR: 0.90;95%CI: 0.50, 1.64) (Table 3).

**Table 2:** Surviving sepsis campaign 3-hour bundle time to completion and adherence.

	All Cases (n = 1191)	Admitted to Local Hospital (n = 832)	Transferred (n = 359)	Difference (95% CI)
Primary Outcome	Median (IQR)	Median (IQR)	Median (IQR)	
Time to completion of 3-Hr bundle adherence (min)	52 (26–102)	50 (26–98)	55 (26–116)	−4 (−14, 3)^a^
3-Hr bundle adherence	164 (13.8)	120 (14.4)	44 (12.3)	2.2 (−2.2, 6.5)
**Secondary Outcomes (Dichotomous)**	**n (%)**	**n (%)**	**n (%)**	
Appropriate antibiotics <3 hrs	263 (22.1)	166 (20.0)	97 (27.0)	−7.1 (−12.6, −1.5)
Blood culture <3 hrs	878 (73.7)	636 (76.4)	242 (67.4)	9.0 (3.2, 14.9)
Lactate <3 hrs	1031 (86.6)	725 (87.1)	306 (85.2)	1.9 (−2.6, 6.4)
***Among Bolus Eligible***	**n = 218**	**n = 115**	**n = 103**	
Fluid in less than 3 hrs	68 (31.2)	44 (38.3)	24 (23.3)	15.0 (2.0, 27.9)
In-hospital mortality	112 (9.6)	71 (8.7)	41 (11.6)	−2.9 (−6.9, 1.1)
**Secondary Outcome (Numerical)**	**Median** **(IQR)**	**Median** **(IQR)**	**Median** **(IQR)**	
ED length-of-stay (hours)	2.7 (2.0–3.6)	2.6 (1.9–3.4)	3.1 (2.3–4.0)	−0.6 (−0.7, −0.4)^a^

^a^Brown-Mood median test.

**Table 3:** Multivariate model analysis of SSC 3-hour bundle adherence outcomes and ED LOS among transferred patients compared to locally admitted patients.

Variable	Unadjusted HR (95% CI)	Adjusted HR (95% CI)^a^
3-Hr bundle adherence	0.69 (0.48, 0.980)	0.90 (0.50, 1.64)
**Variable**	**Unadjusted OR (95% CI)**	**Adjusted OR (95% CI)** ^**a**^
Appropriate antibiotics <3 hrs	1.49 (1.11, 1.98)	1.37 (0.81, 2.33)
Blood culture <3 hrs	0.64 (0.49, 0.84)	1.20 (0.72, 2.00)
Lactate <3 hrs	0.85 (0.60, 1.22)	0.69 (0.36, 1.34)^b^
Any antibiotics <3 hrs	1.15 (0.86, 1.54)	1.86 (1.07, 3.23)
*Among Bolus Eligible*		
Fluid in less than 3 hrs	0.49 (0.27, 0.89)	0.38 (0.12, 1.27)
In-hospital mortality	1.38 (0.92, 2.07)	1.18 (0.56, 2.44)^b^
**Variable**	**Unadjusted beta (95% CI)**	**Adjusted beta (95% CI)** ^**a**^
*Linear Regression Model* ED length of stay (hours)	0.56 (0.34, 0.79)	0.91 (0.51, 1.32)
*Quantile Regression Model – Median* ED length of stay (hours)	0.53 (0.37, 0.82)	0.78 (0.39, 0.99)

^a^Models were adjusted for telemedicine, age, sex, total SOFA score, ED infection source, surgery, ED provider type, mode of arrival, comorbidities (COPD, cirrhosis, hypertension, chronic dialysis), triage systolic BP, triage temperature, use of vasopressor in the ED, and mechanical ventilation in ED

^b^Results come from the penalized Firth logistic regression.

Among individual components of bundle adherence, there was no significant difference between transferred and locally admitted patients in receipt of appropriate antibiotics (aOR: 1.37; 95%CI: 0.81, 2.33), blood culture completion (aOR: 1.20, 95%CI: 0.72, 2.00), lactate measurement (aOR: 0.69, 95%CI: 0.36, 1.34) or fluid administration (aOR: 0.38, 95%CI: 0.12, 1.27) (Table 3). However, compared to locally admitted patients, antibiotic administration was greater (aOR: 1.86, 95%CI: 1.07, 3.23).

### In-hospital mortality and ED LOS

There was no difference in in-hospital mortality between the two groups in bivariate analysis (12% transferred vs 9% non-transferred; difference 2.9%; 95% CI: [−6.9], 1.1%). Final adjusted models further indicated no difference in mortality (aOR: 1.18; 95%CI: 0.56, 2.44). Transferred patients had a longer ED length-of-stay (3.1 hours vs 2.6 hours; difference 0.6 hours; 95%CI: 0.4, 0.7) (Table 2). These results persisted in both the linear regression model (β: 0.91; 95%CI: 0.51–1.32) and quantile regression model (β: 0.78; 95%CI: 0.39–0.99).

### Subgroup analysis

Stratified analysis by transfer timing among the 359 that were transferred showed that 50% were transferred within 1.6 hours. There was no difference in 3-hour bundle adherence observed between the two groups (13% of those transferred within 1.6 hours vs. 12% of those transferred > 1.6 hours; difference 0.7%; 95%CI: [−6.7], 8.1%).

## Discussion

In this study of rural ED sepsis care, we found no significant differences in SSC bundle adherence or timing among transferred and locally admitted rural sepsis patients, but there were differences observed in specific bundle elements. This finding was unexpected, because prior studies have shown that patients undergoing interhospital transfer have lower adherence to SSC guidelines and that interhospital transfer is associated with delays in receiving early sepsis care [12,18,36]. Furthermore, several studies have shown that care in a high-volume hospital is associated with better outcomes, which we hypothesized was related to quality of early treatment [11,14,37]. Thus, the results of our study, although less biased than prior studies, contradicts previous research in this area.

Early aggressive intervention has been shown to improve outcomes in sepsis. Several prior studies have shown that rural sepsis patients may be less likely to receive guideline-adherent care, [37,38] and transfer may further contribute to delays [18]. Overall, in our study of Midwestern rural EDs, we found relatively low sepsis guideline adherence, which could be from sepsis under-recognition or individualized care recommendations. The principal reason for low adherence in our cohort was antibiotic timing and selection, and more recent versions of the SSC guidelines have acknowledged that narrow source-directed therapy may at times be appropriate [39]. Whereas prior work compared rural transferred patients to patients treated in tertiary care centers, [18] a strength of our work is that it isolated the process of interhospital transfer by comparing transferred sepsis patients to locally admitted rural sepsis cases. Furthermore, both groups were comprised of patients treated by the same providers in the same hospitals, which improves the internal validity and limits overall bias in our study design.

Our bivariate analysis also highlights opportunities for improvement. The timing and appropriateness of antibiotic administration are both important in early sepsis care, but administration of appropriate early antibiotics was low. Transferred patients were more likely to receive antibiotics prior to transfer, suggesting that communication with tertiary hospitals during the process of arranging transfer may have contributed to greater sepsis recognition and more timely antibiotic administration. Another area of early sepsis care identified that could be improved was fluid resuscitation. Early and adequate fluid resuscitation has previously been shown to improve outcomes among sepsis patients [40]. A proportion of patients in our study did not complete the fluids component of the bundle, so improving the timing of fluid administration in those who qualify may provide an opportunity to improve patient outcomes. Although transferred patients had higher illness severity, our adjusted models incorporated multiple severity indicators, and the divergence in early antibiotics versus fluids was not fully explained by these factors. This suggests that workflow and logistical elements related to the transfer process may influence treatment timing, and residual confounding by unmeasured severity-related factors remains possible.

Our study has a few important limitations. First, this was a retrospective study, which presents the possibility of unmeasured confounders influencing the outcome. However, since our primary outcome was not a clinical outcome but rather a process outcome, it is likely that internal validity was maintained. Second, we used complete-case analysis, which may introduce selection bias if the excluded cases differ systematically from those included; however, missingness in our dataset was limited and occurred primarily in non-outcome variables, making substantial bias unlikely. Data abstraction was performed using manual chart abstraction, which provides a source of misclassification error. However, internal validity was maintained by periodically performing rigorous training and quality checks. We also note that patients transferred outside the participating network were excluded because detailed treatment data were unavailable. These patients may differ from included transferred patients, introducing possible selection bias and limiting generalizability to similar rural transfer networks. Last, our study was conducted using patients who received initial care in rural or low-volume hospitals in a single health system that participated in aggressive sepsis training and quality improvement. Therefore these findings may have limited generalizability in other networks.

## Conclusion

In this study we found that for rural sepsis patients interhospital transfer was not an independent risk factor for delayed time-to-completion of the surviving sepsis campaign bundle. Furthermore in our study population we observed no association between interhospital transfer and reduced adherence with sepsis care guidelines. Future work should focus on alternative methods for increasing adherence with sepsis guidelines in rural hospitals.
