# Supplementary material for: The association between interhospital transfer and sepsis guideline adherence among rural emergency department patients
Source: PLoS One. 2026 Jul 10;21(7):e0351133. doi: 10.1371/journal.pone.0351133 (PMC13353942; doi:10.1371/journal.pone.0351133)
Supplement: Attachment — Submitted filename: PLOSONE_Response to Reviewers.docx [file pone.0351133.s001.docx]

Sarah Jose, PhD

Roy J. and Lucille A.
Carver College of Medicine

*Cole Wymore, BS*

*Medical Student*

Department of Emergency Medicine

200 Hawkins Drive, GH SE203

Iowa City, Iowa 52242

Web	Cole-wymore@uiowa.edu

Staff Editor, PLOS ONE

RE: Manuscript Revision: **PONE-D-25-02566**

Dear Dr. Jose:

Thank you for the opportunity to revise our manuscript, entitled “The Association Between Interhospital Transfer and High-Quality Rural Sepsis Emergency Department Care.” We are submitting our revision incorporating the comments that your reviewers provided through the review process, and we would like to thank the reviewers for their comments that have helped us improve the quality of the manuscript.

***Note:*** Comments by the Editors and Reviewers are numbered. Responses appear below each comment. Text from the manuscript is indented and additions appear in italics, where applicable. In the submitted manuscript, changes have been tracked. Thank you for the opportunity to improve our manuscript.

***Journal Requirements:***

**1. Please ensure that your manuscript meets PLOS ONE's style requirements, including those for file naming. The PLOS ONE style templates can be found at**

**Response:** The manuscript has been updated to meet PLOS ONE’s style requirements including those for file naming.

**2. Thank you for stating the following financial disclosure:**

**[This study was funded by the Agency for Healthcare Research and Quality (K08HS025753) and the Institute for Clinical and Translational Science at the University of Iowa through a grant from the National Center for Advancing Translational Sciences at the National Institutes of Health (UL1TR002537). Dr. Mohr is additionally supported by funding from the Rural Telehealth Research Center with funding from the Health Resources and Services Administration (U3GRH40003).].**

**Please state what role the funders took in the study.  If the funders had no role, please state: "The funders had no role in study design, data collection and analysis, decision to publish, or preparation of the manuscript."**

**If this statement is not correct you must amend it as needed.**

**Please include this amended Role of Funder statement in your cover letter; we will change the online submission form on your behalf.**

**Response:** The funders had no role in the study. Funding statement in manuscript amended to reflect this using requesting statement.

**3. For studies involving third-party data, we encourage authors to share any data specific to their analyses that they can legally distribute. PLOS recognizes, however, that authors may be using third-party data they do not have the rights to share. When third-party data cannot be publicly shared, authors must provide all information necessary for interested researchers to apply to gain access to the data. (**<https://journals.plos.org/plosone/s/data-availability#loc-acceptable-data-access-restrictions>**)**

**For any third-party data that the authors cannot legally distribute, they should include the following information in their Data Availability Statement upon submission:**

1) A description of the data set and the third-party source

2) If applicable, verification of permission to use the data set

3) Confirmation of whether the authors received any special privileges in accessing the data that other researchers would not have

4) All necessary contact information others would need to apply to gain access to the data

**Response:** Data for this study were obtained from Avera eCARE (now Avel eCare). This organization represents a telemedicine network based in Sioux Falls, South Dakota, and serves as a telemedicine hub for a network of over 190 EDs in 13 states, and it is the largest rural ED-based telemedicine network in North America. Permission to use these data were obtained through a data use agreement with Avera eCARE. No additional privileges were received from the group to obtain these data. Individuals may contact Avera eCARE ([contact@avelecare.com](mailto:contact@avelecare.com)) for more information on gaining access to data.

***4. Please amend either the abstract on the online submission form (via Edit Submission) or the abstract in the manuscript so that they are identical.***

**Response:** Revision made so that the abstract is identical on the online submission form and manuscript

***Reviewer #1: Abstract Comments:***

***1. The title can be better stated.***

**Response:** We revised the title of manuscript to more clearly reflect the subject matter in which this study pertains too.

*“The association between interhospital transfer and sepsis guideline adherence among rural emergency department patients”*

***2. The method should be stated precisely, if a standardized model was used, a reference should be given and the method should be*** ***stated.***

**Response:** In the text, we define the models used for each type of outcome (e.g., time-to-event, dichotomous, continuous). We added more citations in the revision for each type of model used.

*“For the time-to-adherence of the SSC 3-hour bundle, we used a Cox proportional hazards model, with right-censoring occurring at 180 minutes [28,29]. For dichotomous outcomes, we used generalized linear models with a binomial distribution and a logit link [30,31]. We used Firth logistic regression as a penalized approach for two dichotomous outcomes due to data sparsity issues [32-34].* *For the continuous outcome of ED LOS, initial Q-Q plots indicated a violation of normality assumption for residuals. We therefore used both linear regression and quantile regression to the median for the continuous response variable [35].”*

***3. The necessity of the study in that it is in a village should be stated with the village statistics and the costs incurred***

**Response:** We are uncertain about what this comment means, and if it is applicable to our study.

***4. The access method and the ethical challenges in the research regarding access should be better explained***
**Response:** We are uncertain about what this comment means, and if it is applicable to our study.

***5. How is the standardization of the trainings?***
**Response:** This study involved no direct provider training. This was an observational study examining the association between interhospital transfer and adherence with the surviving sepsis campaign (SSC) bundle. The SSC bundle guidelines are standardized care protocols for the care of sepsis patients. Therefore, every emergency department provider is expected to know and implement this protocol when caring for sepsis patients as it is considered standard-of-care. For training pertaining to data abstraction, all abstractors received formal and standardized training on how to complete the data abstraction form in REDCap.

***6. How was the distribution of patients in the two groups in the method?***
**Response:** Since this was a retrospective, observational study the distribution of patient into the two groups (locally admitted patients vs patients undergoing interhospital transfer) was dependent on the ED providers decision. This study simply observed ED sepsis patients during a several year period and stratified this group based on being admitted from the ED to the hospital or being transferred from the ED to another hospital before being admitted. The distribution of characteristics within each group is presented in Table 1.

***7. Was the standardization method carried out in all hospital departments or was it different?***
**Response:** This may not apply to our study. If this question is referring to the surviving sepsis campaign bundle (lactate measurement, fluid resuscitation, antibiotics, blood cultures) then yes, this was carried out in all emergency departments as it is considered standard of care for sepsis patients.

***8. Ethical considerations should be explained in a separate paragraph at the end of the article above the conflict of interest.***
**Response:** This does not apply to our study as this was a retrospective cohort study of previously collected data from electronic health records.

***9. If there are no considerations, the names of the pre-hospital emergency responders should be included in a table with the number of patients.***
**Response:** This comment does not apply to our study as we did not examine any aspect of pre-hospital emergency care.

***10. You can specify the inclusion and exclusion criteria in the method precisely.***
**Response:** The inclusion criteria for participants was described in the methods section. Patients with sepsis seen in a participating rural emergency were included. The definition for rural emergency department was defined. The definition of sepsis used in the study was also defined. It is difficult to condense this as the definition of sepsis used required participants to meet multiple criteria. The exclusion criteria is clearly stated as participants who were transferred out of the participating hospital network due to missing data. Additionally, the inclusion and exclusion criteria with their associated numbers are identified in Figure 1 flowchart.

*“Participants included all adult (age ≥ 18 years) sepsis patients seen in a participating rural ED during the study period. Rural EDs were defined by being in a county designated as rural by the Health Resources and Services Administration [23]. Sepsis was defined as requiring all of the following criteria: 1) inpatient discharge diagnosis of sepsis based on International Classification of Diseases, 10th edition, Clinical Modification (ICD-10-CM) codes, 2) infection diagnosed in the ED (based on clinical documentation), 3) organ failure in the ED (defined as a Sequential Organ Failure Assessment Score [SOFA] of 2 or greater or a change of at least 2 in those with chronic disease), and 4) at least 2 systemic inflammatory response syndrome (SIRS) criteria in the ED. Patients who were transferred out of the participating hospital network were excluded for missing data.”*

***11. Conclusions in this study should be made based on the findings.*
Response:**

The conclusion stated in this manuscript were made based on the findings of our study. We stated that interhospital transfer was not an independent risk factor for delayed time-to-completion of the SSC bundle. Additionally, we observed no association between interhospital transfer and reduced adherence with sepsis care guidelines (i.e., SSC bundle). Findings from this study which were included in the results section that support these claims.

*“Transferred and locally admitted patients had similar median time to 3-hour bundle completion (59 vs 51 min; difference 9 min; 95%CI: 3, 18) (Table 2). Completion of the SSC 3-hour bundle was similar between transferred and locally admitted patients (15.6% in both groups). Adjusted for potential confounders, the event rates of SSC 3-hour bundle completion were not significantly different for these groups (aHR: 0.83;95%CI: 0.56, 1.23)”*

***12. Research limitations should be included in this study, and a specific innovation was created to solve the limitations.***

**Response:**

Research limitations were included in the last paragraph of the discussion. Specific innovations to overcome these limitations were also described, i.e., maintained internal validity through abstractor training and quality checks, selected primary outcome that was process outcome rather than clinical outcome.

*“Our study has a few important limitations. First, this was a retrospective study, which presents the possibility of unmeasured confounders influencing the outcome. However, since our primary outcome was not a clinical outcome but rather a process outcome, it is likely that internal validity was maintained. Second, some data could be incorrectly or incompletely recorded. The variables used in our analysis were selected because they were likely to be recorded correctly in electronic health records. Third, data abstraction was performed using manual chart abstraction, which provides a source of misclassification error. However, internal validity was* *maintained by periodically performing rigorous training and quality checks. Last, our study was conducted using patients who received initial care in rural or low-volume hospitals in a single health system that participated in aggressive sepsis training and quality improvement. Therefore, these findings may have limited generalizability in other networks.”*

Thank you for the time you took in helping us to improve our manuscript. We think your comments have clearly made our paper stronger. We continue to think that this topic is particularly important, and we appreciate your willingness to consider our manuscript for publication.

Sincerely,

Cole Wymore, BS

Medical Student, Department of Emergency Medicine

University of Iowa Carver College of Medicine
