# Supplementary material for: The association between interhospital transfer and sepsis guideline adherence among rural emergency department patients
Source: PLoS One. 2026 Jul 10;21(7):e0351133. doi: 10.1371/journal.pone.0351133 (PMC13353942; doi:10.1371/journal.pone.0351133)
Supplement: Attachment — Submitted filename: Reviewer comments_K.K.Owusu.docx [file pone.0351133.s002.docx]

Feedback on:

**The Association Between Interhospital Transfer and High-Quality Rural Sepsis Emergency Department Care**

**Summary of the research and your overall impression**

This manuscript evaluates whether interhospital transfer is associated with delayed completion of the Surviving Sepsis Campaign (SSC) 3-hour bundle among rural emergency department (ED) patients. Using a retrospective secondary analysis of a multicenter telemedicine network cohort, the authors compare transferred patients with locally admitted patients with sepsis. The primary outcome is complete adherence to the SSC 3-hour bundle; secondary outcomes include bundle components, ED length of stay, and in-hospital mortality.

The study addresses an important and clinically relevant question in rural emergency care. The study design and statistical methods are generally appropriate for the research question. The methods are largely reproducible but require additional information on missing-data handling. The data also support the primary conclusion that transfer was not independently associated with delayed bundle completion. Waiver of consent is appropriately reported. No ethical concerns are evident.

**Major Issues:**

- There was a significantly higher telemedicine use among transferred patients, therefore, the authors may need to clarify if telemedicine was included in the adjusted models and discuss possible effects of telemedicine, including on antibiotic administration
- The authors should consider addressing potential bias introduced by the exclusion of incomplete records
- For the finding that transferred patients were more likely to receive antibiotics but less likely to receive fluids, the authors may need to clarify whether illness severity fully explains this divergence
- Patients transferred outside the participating hospital network were excluded due to missing data; the authors may have to consider discussing how these patients might differ from the included transfers and also whether their exclusion may limit generalizability or introduce selection bias.

**Minor Issues:**

- Consider revising the sentence in Line 61, including the spelling of “receiving”**,** to improve readability
- Consider revising the sentence in Line 81, including the spelling of “acsessed”**,** to improve readability
- Typographical errors- such as “Campign” (Table 2 heading, line 196), “blundle” (line 126) should be corrected

**Comments to Editor**

The study appears methodologically sound and clinically relevant. The requested revisions focus primarily on clarifying methodological decisions rather than fundamental flaws. Provided these issues are adequately addressed, the manuscript would likely meet PLOS ONE’s criteria for technical soundness and transparency.
