# Supplementary material for: The association between interhospital transfer and sepsis guideline adherence among rural emergency department patients
Source: PLoS One. 2026 Jul 10;21(7):e0351133. doi: 10.1371/journal.pone.0351133 (PMC13353942; doi:10.1371/journal.pone.0351133)
Supplement: Attachment — Submitted filename: Reviewer comments_final.docx [file pone.0351133.s003.docx]

Feedback on:

**The Association Between Interhospital Transfer and High-Quality Rural Sepsis Emergency Department Care**

**Summary of the research and your overall impression**

This manuscript evaluates whether interhospital transfer is associated with delayed completion of the Surviving Sepsis Campaign (SSC) 3-hour bundle among rural emergency department (ED) patients. Using a retrospective secondary analysis of a multicenter telemedicine network cohort, the authors compare transferred patients with locally admitted patients with sepsis. The primary outcome is complete adherence to the SSC 3-hour bundle; secondary outcomes include bundle components, ED length of stay, and in-hospital mortality.

The study addresses an important and clinically relevant question in rural emergency care. The study design and statistical methods are generally appropriate for the research question. The methods are largely reproducible but require additional information on missing-data handling. The data also support the primary conclusion that transfer was not independently associated with delayed bundle completion. Waiver of consent is appropriately reported. No ethical concerns are evident.

**Major Issues:**

1. **There was a significantly higher telemedicine use among transferred patients, therefore, the authors may need to clarify if telemedicine was included in the adjusted models and discuss possible effects of telemedicine, including on antibiotic administration**

**Response:** We thank the reviewer for this insightful comment. Telemedicine use has now been included as a covariate in all adjusted models to account for its potential confounding effects. We have updated the Results section accordingly. We also expanded the Discussion to address the possible influence of telemedicine on early sepsis care processes, including its potential role in improving antibiotic administration.

***“****Although transferred patients had higher illness severity, our adjusted models incorporated multiple severity indicators, and the divergence in early antibiotics versus fluids was not fully explained by these factors. This suggests that workflow and logistical elements related to the transfer process may influence treatment timing, and residual confounding by unmeasured severity-related factors remains possible.”*

2. **The authors should consider addressing potential bias introduced by the exclusion of incomplete records.**

**Response:** Thank you for raising this important point. We agree that excluding incomplete records can introduce the potential for selection bias. In the TELEVISED dataset, missingness primarily occurred in variables not essential for determining exposure or outcomes, and incomplete cases represented a small proportion of the overall cohort. Because the pattern of missingness was largely unrelated to outcomes, complete‑case analysis was deemed the most appropriate approach. We have added text to the Methods and Discussion sections to acknowledge this limitation and its possible implications.

*“We used complete‑case analysis, which may introduce selection bias if the excluded cases differ systematically from those included; however, missingness in our dataset was limited and occurred primarily in non‑outcome variables, making substantial bias unlikely.”*

3. **For the finding that transferred patients were more likely to receive antibiotics but less likely to receive fluids, the authors may need to clarify whether illness severity fully explains this divergence**

**Response:** Thank you for this thoughtful comment. Our adjusted models already include several indicators of illness severity and acuity at presentation, including total SOFA score, triage vital signs, vasopressor use in the ED, mechanical ventilation in the ED, and infection source. After accounting for these factors, transferred patients still demonstrated higher odds of receiving any antibiotics within 3 hours, while no significant difference was observed for early fluid administration among bolus-eligible patients. These findings suggest that measured severity does not fully explain the observed divergence between antibiotics and fluids. We have clarified this in the Discussion and now note that residual unmeasured factors related to transfer logistics, workflow prioritization, or clinical decision-making may contribute to these patterns.

*“Although transferred patients had higher illness severity, our adjusted models incorporated multiple severity indicators, and the divergence in early antibiotics versus fluids was not fully explained by these factors. This suggests that workflow and logistical elements related to the transfer process may influence treatment timing. Residual confounding by unmeasured severity-related factors is still possible.”*

4. **Patients transferred outside the participating hospital network were excluded due to missing data; the authors may have to consider discussing how these patients might differ from the included transfers and also whether their exclusion may limit generalizability or introduce selection bias.**

**Response:** Thank you for this thoughtful comment. Patients transferred outside the participating hospital network were excluded because essential clinical and treatment data were not available for those encounters. We recognize that these patients may differ systematically from included transferred patients, particularly with regard to illness severity, transfer distance, or hospital capabilities. Their exclusion may therefore introduce selection bias and limits the generalizability of our findings to transfers occurring within similar integrated rural networks. We have added text to the Discussion acknowledging this potential limitation.

*“We also note that patients transferred outside the participating network were excluded because detailed treatment data were unavailable. These patients may differ from included transferred patients, introducing possible selection bias and limiting generalizability to similar rural transfer networks.”*

**Minor Issues:**

5. **Consider revising the sentence in Line 61, including the spelling of “receiving”, to improve readability**
6. **Consider revising the sentence in Line 81, including the spelling of “acsessed”, to improve readability**
7. **Typographical errors- such as “Campign” (Table 2 heading, line 196), “blundle” (line 126) should be corrected**

**Response:** The minor issues (5-7) have been revised.

**Final:**

8. **The study appears methodologically sound and clinically relevant. The requested revisions focus primarily on clarifying methodological decisions rather than fundamental flaws. Provided these issues are adequately addressed, the manuscript would likely meet PLOS ONE’s criteria for technical soundness and transparency.**

**Response:** We thank the reviewer for their time in appraising our study.
