# Supplementary material for: The association between interhospital transfer and sepsis guideline adherence among rural emergency department patients
Source: PLoS One. 2026 Jul 10;21(7):e0351133. doi: 10.1371/journal.pone.0351133 (PMC13353942; doi:10.1371/journal.pone.0351133)
Supplement: Peer review history

# Author comment: Author response to Decision Letter 0

**DOI:** 10.1371/journal.pone.0351133.r001

**Transfer Alert**

This paper was transferred from another journal. As a result, its full editorial history (including decision letters, peer reviews and author responses) may not be present.

5 Feb 2025

# Peer review: Decision Letter 0

**DOI:** 10.1371/journal.pone.0351133.r002

**Authors:** Sarah Jose

13 Jul 2025

PONE-D-25-02566

The Association Between Interhospital Transfer and High-Quality Rural Sepsis Emergency Department Care

PLOS ONE

Dear Dr. Wymore,

Thank you for submitting your manuscript to PLOS ONE. After careful consideration, we feel that it has merit but does not fully meet PLOS ONE’s publication criteria as it currently stands. Therefore, we invite you to submit a revised version of the manuscript that addresses the points raised during the review process.

Please note that we have only been able to secure a single reviewer to assess your manuscript. We are issuing a decision on your manuscript at this point to prevent further delays in the evaluation of your manuscript. Please be aware that the editor who handles your revised manuscript might find it necessary to invite additional reviewers to assess this work once the revised manuscript is submitted. However, we will aim to proceed on the basis of this single review if possible.

Please review the reviewer's recommendations and revise your manuscript accordingly. They particularly focus on improving the methods and reporting. Upon resubmission, please provide a point-by-point response to each reviewer comment.

Please submit your revised manuscript by Aug 21 2025 11:59PM. If you will need more time than this to complete your revisions, please reply to this message or contact the journal office at plosone@plos.org. When you're ready to submit your revision, log on to https://www.editorialmanager.com/pone/ and select the 'Submissions Needing Revision' folder to locate your manuscript file.

Please include the following items when submitting your revised manuscript:

- A rebuttal letter that responds to each point raised by the academic editor and reviewer(s). You should upload this letter as a separate file labeled 'Response to Reviewers'.
- A marked-up copy of your manuscript that highlights changes made to the original version. You should upload this as a separate file labeled 'Revised Manuscript with Track Changes'.
- An unmarked version of your revised paper without tracked changes. You should upload this as a separate file labeled 'Manuscript'.

If you would like to make changes to your financial disclosure, please include your updated statement in your cover letter. Guidelines for resubmitting your figure files are available below the reviewer comments at the end of this letter.

If applicable, we recommend that you deposit your laboratory protocols in protocols.io to enhance the reproducibility of your results. Protocols.io assigns your protocol its own identifier (DOI) so that it can be cited independently in the future. For instructions see: https://journals.plos.org/plosone/s/submission-guidelines#loc-laboratory-protocols. Additionally, PLOS ONE offers an option for publishing peer-reviewed Lab Protocol articles, which describe protocols hosted on protocols.io. Read more information on sharing protocols at https://plos.org/protocols?utm_medium=editorial-email&utm_source=authorletters&utm_campaign=protocols.

We look forward to receiving your revised manuscript.

Kind regards,

Sarah Jose, Ph.D.

Staff Editor

PLOS ONE

Journal requirements:

When submitting your revision, we need you to address these additional requirements.

1. Please ensure that your manuscript meets PLOS ONE's style requirements, including those for file naming. The PLOS ONE style templates can be found at

https://journals.plos.org/plosone/s/file?id=wjVg/PLOSOne_formatting_sample_main_body.pdf and

https://journals.plos.org/plosone/s/file?id=ba62/PLOSOne_formatting_sample_title_authors_affiliations.pdf

2. Thank you for stating the following financial disclosure:

[This study was funded by the Agency for Healthcare Research and Quality (K08HS025753) and the Institute for Clinical and Translational Science at the University of Iowa through a grant from the National Center for Advancing Translational Sciences at the National Institutes of Health (UL1TR002537). Dr. Mohr is additionally supported by funding from the Rural Telehealth Research Center with funding from the Health Resources and Services Administration (U3GRH40003).].

Please state what role the funders took in the study.  If the funders had no role, please state: "The funders had no role in study design, data collection and analysis, decision to publish, or preparation of the manuscript."

If this statement is not correct you must amend it as needed.

Please include this amended Role of Funder statement in your cover letter; we will change the online submission form on your behalf.

3. For studies involving third-party data, we encourage authors to share any data specific to their analyses that they can legally distribute. PLOS recognizes, however, that authors may be using third-party data they do not have the rights to share. When third-party data cannot be publicly shared, authors must provide all information necessary for interested researchers to apply to gain access to the data. (https://journals.plos.org/plosone/s/data-availability#loc-acceptable-data-access-restrictions)

For any third-party data that the authors cannot legally distribute, they should include the following information in their Data Availability Statement upon submission:

1) A description of the data set and the third-party source

2) If applicable, verification of permission to use the data set

3) Confirmation of whether the authors received any special privileges in accessing the data that other researchers would not have

4) All necessary contact information others would need to apply to gain access to the data

4. Please amend either the abstract on the online submission form (via Edit Submission) or the abstract in the manuscript so that they are identical.

Additional Editor Comments (if provided):

[Note: HTML markup is below. Please do not edit.]

Reviewers' comments:

Reviewer's Responses to Questions

**Comments to the Author**

1. Is the manuscript technically sound, and do the data support the conclusions?

The manuscript must describe a technically sound piece of scientific research with data that supports the conclusions. Experiments must have been conducted rigorously, with appropriate controls, replication, and sample sizes. The conclusions must be drawn appropriately based on the data presented.

Reviewer #1: Partly

**********

2. Has the statistical analysis been performed appropriately and rigorously?

Reviewer #1: No

**********

3. Have the authors made all data underlying the findings in their manuscript fully available?

The PLOS Data policy requires authors to make all data underlying the findings described in their manuscript fully available without restriction, with rare exception (please refer to the Data Availability Statement in the manuscript PDF file). The data should be provided as part of the manuscript or its supporting information, or deposited to a public repository. For example, in addition to summary statistics, the data points behind means, medians and variance measures should be available. If there are restrictions on publicly sharing data—e.g. participant privacy or use of data from a third party—those must be specified.

Reviewer #1: Yes

**********

4. Is the manuscript presented in an intelligible fashion and written in standard English?

PLOS ONE does not copyedit accepted manuscripts, so the language in submitted articles must be clear, correct, and unambiguous. Any typographical or grammatical errors should be corrected at revision, so please note any specific errors here.

Reviewer #1: Yes

**********

5. Review Comments to the Author

Please use the space provided to explain your answers to the questions above. You may also include additional comments for the author, including concerns about dual publication, research ethics, or publication ethics. (Please upload your review as an attachment if it exceeds 20,000 characters)

Reviewer #1: Dear authors,

In addition to thanking you for the research that has been done, the following topics can be helpful as suggestions in the article:

Abstract

The title can be better stated

The method should be stated precisely, if a standardized model was used, a reference should be given and the method should be stated

The necessity of the study in that it is in a village should be stated with the village statistics and the costs incurred

The access method and the ethical challenges in the research regarding access should be better explained

How is the standardization of the trainings?

How was the distribution of patients in the two groups in the method?

Was the standardization method carried out in all hospital departments or was it different?

Ethical considerations should be explained in a separate paragraph at the end of the article above the conflict of interest.

If there are no considerations, the names of the pre-hospital emergency responders should be included in a table with the number of patients.

You can specify the inclusion and exclusion criteria in the method precisely.

Conclusions in this study should be made based on the findings.

Research limitations should be included in this study and a specific innovation was created to solve the limitations.

**********

6. PLOS authors have the option to publish the peer review history of their article (what does this mean?). If published, this will include your full peer review and any attached files.

If you choose “no”, your identity will remain anonymous but your review may still be made public.

**Do you want your identity to be public for this peer review?**  For information about this choice, including consent withdrawal, please see our Privacy Policy.

Reviewer #1: No

**********

[NOTE: If reviewer comments were submitted as an attachment file, they will be attached to this email and accessible via the submission site. Please log into your account, locate the manuscript record, and check for the action link "View Attachments". If this link does not appear, there are no attachment files.]

While revising your submission, please upload your figure files to the Preflight Analysis and Conversion Engine (PACE) digital diagnostic tool, https://pacev2.apexcovantage.com/. PACE helps ensure that figures meet PLOS requirements. To use PACE, you must first register as a user. Registration is free. Then, login and navigate to the UPLOAD tab, where you will find detailed instructions on how to use the tool. If you encounter any issues or have any questions when using PACE, please email PLOS at figures@plos.org. Please note that Supporting Information files do not need this step.

# Author comment: Author response to Decision Letter 1

**DOI:** 10.1371/journal.pone.0351133.r003

25 Oct 2025

Dear Reviewer and Editor,

Thank you for considering our manuscript, "The association between interhospital transfer and sepsis guideline adherence among rural emergency department patients," for publication. Our responses to the reviewer comments have been included in the "response to reviewer" document.

Thanks,

Cole Wymore

Attachment Submitted filename: PLOSONE_Response to Reviewers.docx

# Peer review: Decision Letter 1

**DOI:** 10.1371/journal.pone.0351133.r004

**Authors:** Ramya Iyadurai

15 Feb 2026

PONE-D-25-02566R1

The Association Between Interhospital Transfer and Sepsis Guideline Adherence Among Rural Emergency Department Patients

PLOS One

Dear Dr. Wymore,

Thank you for submitting your manuscript to PLOS ONE. After careful consideration, we feel that it has merit but does not fully meet PLOS ONE’s publication criteria as it currently stands. Therefore, we invite you to submit a revised version of the manuscript that addresses the points raised during the review process.

Kindly address all the reviewer’s suggestions with a focus on these issues , address issues regarding bias arising from exclusion of patients with missing data, and use of telemedicine in the patient groups.

Please submit your revised manuscript by Apr 01 2026 11:59PM. If you will need more time than this to complete your revisions, please reply to this message or contact the journal office at plosone@plos.org. When you're ready to submit your revision, log on to https://www.editorialmanager.com/pone/ and select the 'Submissions Needing Revision' folder to locate your manuscript file.

Please include the following items when submitting your revised manuscript:

- A letter that responds to each point raised by the academic editor and reviewer(s). You should upload this letter as a separate file labeled 'Response to Reviewers'.
- A marked-up copy of your manuscript that highlights changes made to the original version. You should upload this as a separate file labeled 'Revised Manuscript with Track Changes'.
- An unmarked version of your revised paper without tracked changes. You should upload this as a separate file labeled 'Manuscript'.

-->If you would like to make changes to your financial disclosure, please include your updated statement in your cover letter. Guidelines for resubmitting your figure files are available below the reviewer comments at the end of this letter.

If applicable, we recommend that you deposit your laboratory protocols in protocols.io to enhance the reproducibility of your results. Protocols.io assigns your protocol its own identifier (DOI) so that it can be cited independently in the future. For instructions see: https://journals.plos.org/plosone/s/submission-guidelines#loc-laboratory-protocols. Additionally, PLOS ONE offers an option for publishing peer-reviewed Lab Protocol articles, which describe protocols hosted on protocols.io. Read more information on sharing protocols at https://plos.org/protocols?utm_medium=editorial-email&utm_source=authorletters&utm_campaign=protocols.

We look forward to receiving your revised manuscript.

Kind regards,

Ramya Iyadurai

Academic Editor

PLOS One

Journal Requirements:

If the reviewer comments include a recommendation to cite specific previously published works, please review and evaluate these publications to determine whether they are relevant and should be cited. There is no requirement to cite these works unless the editor has indicated otherwise.

Please review your reference list to ensure that it is complete and correct. If you have cited papers that have been retracted, please include the rationale for doing so in the manuscript text, or remove these references and replace them with relevant current references. Any changes to the reference list should be mentioned in the rebuttal letter that accompanies your revised manuscript. If you need to cite a retracted article, indicate the article’s retracted status in the References list and also include a citation and full reference for the retraction notice.

Additional Editor Comments (if provided):

Kindly address all the reviewers suggestions

[Note: HTML markup is below. Please do not edit.]

Reviewers' comments:

Reviewer's Responses to Questions

**Comments to the Author**

1. If the authors have adequately addressed your comments raised in a previous round of review and you feel that this manuscript is now acceptable for publication, you may indicate that here to bypass the “Comments to the Author” section, enter your conflict of interest statement in the “Confidential to Editor” section, and submit your "Accept" recommendation.

Reviewer #1: (No Response)

Reviewer #2: (No Response)

**********

2. Is the manuscript technically sound, and do the data support the conclusions?

The manuscript must describe a technically sound piece of scientific research with data that supports the conclusions. Experiments must have been conducted rigorously, with appropriate controls, replication, and sample sizes. The conclusions must be drawn appropriately based on the data presented.

Reviewer #1: No

Reviewer #2: Yes

**********

3. Has the statistical analysis been performed appropriately and rigorously?

Reviewer #1: No

Reviewer #2: Yes

**********

4. Have the authors made all data underlying the findings in their manuscript fully available?

The PLOS Data policy requires authors to make all data underlying the findings described in their manuscript fully available without restriction, with rare exception (please refer to the Data Availability Statement in the manuscript PDF file). The data should be provided as part of the manuscript or its supporting information, or deposited to a public repository. For example, in addition to summary statistics, the data points behind means, medians and variance measures should be available. If there are restrictions on publicly sharing data—e.g. participant privacy or use of data from a third party—those must be specified.

Reviewer #1: No

Reviewer #2: Yes

**********

5. Is the manuscript presented in an intelligible fashion and written in standard English?

PLOS ONE does not copyedit accepted manuscripts, so the language in submitted articles must be clear, correct, and unambiguous. Any typographical or grammatical errors should be corrected at revision, so please note any specific errors here.

Reviewer #1: Yes

Reviewer #2: Yes

**********

6. Review Comments to the Author

Please use the space provided to explain your answers to the questions above. You may also include additional comments for the author, including concerns about dual publication, research ethics, or publication ethics. (Please upload your review as an attachment if it exceeds 20,000 characters)

Reviewer #1: Dear Authors,

Thank you for the corrections made

The following have been corrected during the review

The title and abstract have been corrected

The topics and changes requested in the text have not been answered. Please highlight the changes.

Reviewer #2: Summary of the research and your overall impression:

This manuscript evaluates whether interhospital transfer is associated with delayed completion of the Surviving Sepsis Campaign (SSC) 3-hour bundle among rural emergency department (ED) patients. Using a retrospective secondary analysis of a multicenter telemedicine network cohort, the authors compare transferred patients with locally admitted patients with sepsis. The primary outcome is complete adherence to the SSC 3-hour bundle; secondary outcomes include bundle components, ED length of stay, and in-hospital mortality.

The study addresses an important and clinically relevant question in rural emergency care. The study design and statistical methods are generally appropriate for the research question. The methods are largely reproducible but require additional information on missing-data handling. The data also support the primary conclusion that transfer was not independently associated with delayed bundle completion. Waiver of consent is appropriately reported. No ethical concerns are evident.

Major Issues:

• There was a significantly higher telemedicine use among transferred patients, therefore, the authors may need to clarify if telemedicine was included in the adjusted models and discuss possible effects of telemedicine, including on antibiotic administration

• The authors should consider addressing potential bias introduced by the exclusion of incomplete records

• For the finding that transferred patients were more likely to receive antibiotics but less likely to receive fluids, the authors may need to clarify whether illness severity fully explains this divergence

• Patients transferred outside the participating hospital network were excluded due to missing data; the authors may have to consider discussing how these patients might differ from the included transfers and also whether their exclusion may limit generalizability or introduce selection bias.

Minor Issues:

• Consider revising the sentence in Line 61, including the spelling of “receiving”, to improve readability

• Consider revising the sentence in Line 81, including the spelling of “acsessed”, to improve readability

• Typographical errors- such as “Campign” (Table 2 heading, line 196), “blundle” (line 126) should be corrected

**********

7. PLOS authors have the option to publish the peer review history of their article (what does this mean?). If published, this will include your full peer review and any attached files.

If you choose “no”, your identity will remain anonymous but your review may still be made public.

**Do you want your identity to be public for this peer review?**  For information about this choice, including consent withdrawal, please see our Privacy Policy.

Reviewer #1: No

Reviewer #2: No

**********

[NOTE: If reviewer comments were submitted as an attachment file, they will be attached to this email and accessible via the submission site. Please log into your account, locate the manuscript record, and check for the action link "View Attachments". If this link does not appear, there are no attachment files.]

To ensure your figures meet our technical requirements, please review our figure guidelines: https://journals.plos.org/plosone/s/figures

You may also use PLOS’s free figure tool, NAAS, to help you prepare publication quality figures: https://journals.plos.org/plosone/s/figures#loc-tools-for-figure-preparation.

NAAS will assess whether your figures meet our technical requirements by comparing each figure against our figure specifications.

Attachment Submitted filename: Reviewer comments_K.K.Owusu.docx

# Author comment: Author response to Decision Letter 2

**DOI:** 10.1371/journal.pone.0351133.r005

20 Apr 2026

Please see attached file, "Reviewer comments_final" for response to reviewer and editor comments. Thank you for considering this submission for publication.

Attachment Submitted filename: Reviewer comments_final.docx

# Peer review: Decision Letter 2

**DOI:** 10.1371/journal.pone.0351133.r006

**Authors:** Ramya Iyadurai

25 May 2026

The Association Between Interhospital Transfer and Sepsis Guideline Adherence Among Rural Emergency Department Patients

PONE-D-25-02566R2

Dear Dr. Wymore,

We’re pleased to inform you that your manuscript has been judged scientifically suitable for publication and will be formally accepted for publication once it meets all outstanding technical requirements.

Within one week, you’ll receive an e-mail detailing the required amendments. When these have been addressed, you’ll receive a formal acceptance letter and your manuscript will be scheduled for publication.

An invoice will be generated when your article is formally accepted. Please note, if your institution has a publishing partnership with PLOS and your article meets the relevant criteria, all or part of your publication costs will be covered. Please make sure your user information is up-to-date by logging into Editorial Manager at Editorial Manager® and clicking the ‘Update My Information' link at the top of the page. For questions related to billing, please contact billing support.

If your institution or institutions have a press office, please notify them about your upcoming paper to help maximize its impact. If they’ll be preparing press materials, please inform our press team as soon as possible -- no later than 48 hours after receiving the formal acceptance. Your manuscript will remain under strict press embargo until 2 pm Eastern Time on the date of publication. For more information, please contact onepress@plos.org.

Kind regards,

Ramya Iyadurai

Academic Editor

PLOS One

Additional Editor Comments (optional):

Reviewers' comments:

Reviewer's Responses to Questions

**Comments to the Author**

1. If the authors have adequately addressed your comments raised in a previous round of review and you feel that this manuscript is now acceptable for publication, you may indicate that here to bypass the “Comments to the Author” section, enter your conflict of interest statement in the “Confidential to Editor” section, and submit your "Accept" recommendation.

Reviewer #3: (No Response)

Reviewer #4: (No Response)

**********

2. Is the manuscript technically sound, and do the data support the conclusions?

The manuscript must describe a technically sound piece of scientific research with data that supports the conclusions. Experiments must have been conducted rigorously, with appropriate controls, replication, and sample sizes. The conclusions must be drawn appropriately based on the data presented.

Reviewer #3: Yes

Reviewer #4: Yes

**********

3. Has the statistical analysis been performed appropriately and rigorously?

Reviewer #3: Yes

Reviewer #4: Yes

**********

4. Have the authors made all data underlying the findings in their manuscript fully available?

The PLOS Data policy requires authors to make all data underlying the findings described in their manuscript fully available without restriction, with rare exception (please refer to the Data Availability Statement in the manuscript PDF file). The data should be provided as part of the manuscript or its supporting information, or deposited to a public repository. For example, in addition to summary statistics, the data points behind means, medians and variance measures should be available. If there are restrictions on publicly sharing data—e.g. participant privacy or use of data from a third party—those must be specified.

Reviewer #3: No

Reviewer #4: Yes

**********

5. Is the manuscript presented in an intelligible fashion and written in standard English?

PLOS ONE does not copyedit accepted manuscripts, so the language in submitted articles must be clear, correct, and unambiguous. Any typographical or grammatical errors should be corrected at revision, so please note any specific errors here.

Reviewer #3: Yes

Reviewer #4: Yes

**********

6. Review Comments to the Author

Please use the space provided to explain your answers to the questions above. You may also include additional comments for the author, including concerns about dual publication, research ethics, or publication ethics. (Please upload your review as an attachment if it exceeds 20,000 characters)

Reviewer #3: Thank you for the revised manuscript and for the detailed responses to the previous comments. I think the revision has improved the paper. The added adjustment for telemedicine use and the added discussion of missing data and transfers outside the participating network are helpful.

As a clinician working in a smaller hospital setting, I found the topic relevant to daily practice. In real emergency department work, arranging transfer, communicating with the receiving hospital, giving antibiotics and fluids, and continuing reassessment of the patient often happen at the same time. The manuscript now discusses this practical tension more clearly, especially around antibiotics and fluid resuscitation.

One point still needs clarification. Please make sure the fluid resuscitation result is reported consistently between the submission abstract/metadata and the clean revised manuscript. In the clean manuscript, early fluid administration among bolus-eligible patients appears not statistically significant after adjustment (aOR 0.38; 95% CI 0.12, 1.27). However, the submission abstract reports lower odds of guideline-adherent fluids among transferred patients (aOR 0.37; 95% CI 0.16, 0.88). This discrepancy should be clarified before publication.

It may also be helpful to give the number or proportion of cases excluded because of missing exposure or outcome data. The authors state that missingness was limited, which is reassuring, but a simple number would make this easier for readers to judge.

The data availability statement explains why patient-level data cannot be shared publicly. I understand this for this type of clinical dataset, but I leave it to the journal to confirm whether the restriction is acceptable under the PLOS data policy.

Overall, I think the manuscript is clinically relevant and methodologically acceptable. After these clarifications, I would support publication.

Reviewer #4: The manuscript is technically sound and the main conclusions are broadly supported by the data presented. The study design is appropriate, with adequate sample size in a multicentre setting. The SSC 3hour bundle completion as an outcome is explicitly defined. Internal validity was strengthened by the inclusion of the telemedicine use as a covariate. Given the reported results and adjusted analyses, the conclusion that transfer was not an independent risk factor for delayed bundle completion is consistent with the data presented. The long ED length of stay among transferred patients and other differences are appropriately framed as component-level differences rather than overturning the primary finding.

The scientific language is generally appropriate and consistent with clinical/epidemiological reporting. The methods and results are mostly understandable and logically structured. Terminology is also used correctly and consistently.

Most of the typographical and formatting errors have been resolved, which has improved readability and presentation quality prior to publication. One outstanding is Line 149: “The most common reasons for exclusion was…” Should be: “were”

**********

7. PLOS authors have the option to publish the peer review history of their article (what does this mean?). If published, this will include your full peer review and any attached files.

If you choose “no”, your identity will remain anonymous but your review may still be made public.

**Do you want your identity to be public for this peer review?**  For information about this choice, including consent withdrawal, please see our Privacy Policy.

Reviewer #3: **Yes:**  Dongpo Liu

Reviewer #4: **Yes:**  Dr Busisiwe Msimanga

**********

# Editor report: Acceptance letter

**DOI:** 10.1371/journal.pone.0351133.r007

**Authors:** Ramya Iyadurai

PONE-D-25-02566R2

PLOS One

Dear Dr. Wymore,

I'm pleased to inform you that your manuscript has been deemed suitable for publication in PLOS One. Congratulations! Your manuscript is now being handed over to our production team.

At this stage, our production department will prepare your paper for publication. This includes ensuring the following:

* All references, tables, and figures are properly cited

* All relevant supporting information is included in the manuscript submission,

* There are no issues that prevent the paper from being properly typeset

You will receive further instructions from the production team, including instructions on how to review your proof when it is ready. Please keep in mind that we are working through a large volume of accepted articles, so please give us a few days to review your paper and let you know the next and final steps.

Lastly, if your institution or institutions have a press office, please let them know about your upcoming paper now to help maximize its impact. If they'll be preparing press materials, please inform our press team within the next 48 hours. Your manuscript will remain under strict press embargo until 2 pm Eastern Time on the date of publication. For more information, please contact onepress@plos.org.

You will receive an invoice from PLOS for your publication fee after your manuscript has reached the completed accept phase. If you receive an email requesting payment before acceptance or for any other service, this may be a phishing scheme. Learn how to identify phishing emails and protect your accounts at https://explore.plos.org/phishing.

If we can help with anything else, please email us at customercare@plos.org.

Thank you for submitting your work to PLOS ONE and supporting open access.

Kind regards,

PLOS ONE Editorial Office Staff

on behalf of

Dr. Ramya Iyadurai

Academic Editor

PLOS One
